# Supplementary material for: Are carbon emissions trading and green financial instruments synergistic? -Comprehensive quantitative research based on content analysis
Source: PLoS One. 2024 Mar 7;19(3):e0298601. doi: 10.1371/journal.pone.0298601 (PMC10919601; doi:10.1371/journal.pone.0298601)
Supplement: S1 Data — (ZIP) [file pone.0298601.s001.zip › Data/地级市Z_F工作报告.zip!/║■─╧/╥μ╤⌠╩╨/└┤╫╘╣1⁄2╓┌║┼í╛┬φ┐╦╩2╛▌═°í┐_2010.docx]

各位代表：

　　现在，我代表市人民政府，向大会作政府工作报告，请予审议，并请各位政协委员和其他列席人员提出意见。

一、2009年工作回顾

　　2009年是新一届政府履职的第一年，也是我市发展经受严峻考验的一年。在中共益阳市委的正确领导下，我们坚持以邓小平理论和“三个代表”重要思想为指导，深入贯彻落实科学发展观，积极应对国际金融危机的挑战，及时出台了保增长、保民生、促发展的一系列政策措施，实现了全市经济社会的平稳较快发展，各项指标基本达到年初预期。预计全年完成生产总值550.5亿元，比上年增长12.8%。其中第一产业增加值140亿元，增长6%；第二产业增加值196.4亿元，增长21.2%；第三产业增加值214.1亿元，增长9.7%。完成财政总收入34.07亿元，增长13.3%。完成全社会固定资产投资353亿元，增长36.8%。实现社会消费品零售总额223亿元，增长18.7%。外贸进出口受金融危机影响有所下滑，全年完成进出口总额2.3亿美元，下降10%。城镇居民人均可支配收入13802元，增长10.9%；农民人均纯收入4934元，增长9 %。城镇登记失业率控制在4.5%以内。年末金融机构人民币存贷款余额分别为514.5亿元和266.9亿元，比年初分别增长21%和28.6%。万元生产总值能耗下降4.4%，万元规模工业增加值能耗下降14%。化学需氧量和二氧化硫排放量分别控制在8.8万吨和7.13万吨以下。人口自然增长率为4.95‰。

　　（一）加大企业帮扶力度，工业经济快速增长

　　把保企业作为保增长的重中之重，大力开展“企业服务年”活动，帮助企业渡过难关，全市停产半停产企业由年初的583家减少到34家。特别是采取有效措施缓解企业融资困难，扎实开展“金融促发展、信贷早春行”活动，共签约金额71.58亿元，已发放贷款44.67亿元，全市金融机构全年新增贷款59.7亿元，信贷投放创历史新高，金融支持发展的力度空前加大；加强企业信用担保体系建设，注资1.1亿元入股省、市担保公司，中小企业获得担保贷款2.5亿元；全面推进企业上市工作，8家企业列入省重点上市企业后备资源库。全面清理整顿涉企收费，减轻企业负担2.76亿元。保企业带来了工业经济的快速发展，预计全年完成工业增加值172亿元，比上年增长21.9%。其中规模工业增加值157亿元，增长24%；经济效益综合指数212%，提高20个百分点；规模工业企业达到719家，比上年增加90家；装备制造和食品加工两大产业产值突破百亿元大关。益阳被授牌为“国家高技术产业基地”、“湖南省综合性高技术产业基地”，全年高新技术产业增加值34.7亿元，增长25%。新增湖南名牌8个。益阳高新区被纳入省“千亿园区”发展目标。东部新区建设强力推进，全年完成投资7.6亿元。企业环保设施建设得到加强，全市规模以上工业企业主要污染物排放达标率达到90%以上。

　　（二）狠抓招商引资和项目建设，投资拉动强劲有力

　　把扩大投资作为保增长的根本途径，抓好招商引资和立项争资，强力推进项目建设，促进了投资的快速增长，投资对经济增长的贡献率达8.8个百分点。引资争资成效明显。调整招商引资考核办法，招商引资工作实效增强。精心组织了“2009年湖南·益阳（香港）承接产业转移招商推介会”等活动。引进市域外资金形成固定资产投资131.9亿元；实际利用外资9189万美元，增长14.9%。引进了中核第二三建设公司、台湾宝成集团、大汉集团等一批行业优势企业。抓住国家扩大投资的政策机遇，争取中央新增投资项目672个，争取投资7.52亿元，带动各级财政、企业和社会投资21亿元。全年立项争资70.8亿元，增长28.7%。项目建设扎实推进。大张旗鼓地组织开展了重点工程拆迁、开工、百日劳动竞赛“三项行动”。全市73个重点调度项目完成投资89.5亿元。交通基础设施建设力度加大，完成投资30.1亿元，比上年增长76.5%。益宁城际干道益沧段、S204南茅复线、S202华茅公路茅草街至大通湖段、岳常高速大通湖连接线建成通车，S308西姚公路和黄柘公路、S206桃江至横市公路、益阳至芦林潭千吨级航道、益阳港千吨级码头建设进展顺利，安邵、常安和岳常高速益阳段建设稳步推进，益宁城际干道沧宁段、常安高速桃马连接线、G207益阳段、S225安化段、沙头大桥、黄茅洲大桥、朝阳汽车站相继开工。一批高速公路和铁路的前期工作进展顺利。桃花江核电站建设前期工作基本完成。城市防洪主体工程基本建成。电网建设与改造完成投资5.3亿元。产业类项目超额完成年度投资计划，中联重科及通信网络建设等项目完成投资过亿元。

　　（三）切实加强“三农”工作，农业生产稳步发展

　　扎实抓好“三农”工作，新农村建设稳步推进。认真落实中央各项强农惠农政策，各类政策性补贴得到及时发放。粮食总产238.7万吨，创历史新高。生猪产业健康发展。成功举办了首届中国·湖南（益阳）黑茶文化节暨安化黑茶博览会和大通湖捕捞节、河蟹美食节。安化黑茶成功纳入国家地理标志产品保护名录。现代农业稳步发展，农产品加工规模企业实现增加值20亿元，增长6.3%；市级以上农业产业化龙头企业新增48家，达到222家。农民组织化程度不断提高，全市农民专业合作组织发展到788个。农村土地经营权流转取得新突破，共流转农用地205万亩，其中耕地116万亩，增长131%。现代林业示范市建设不断推进，集体林权制度改革取得新进展。农村基础设施建设进一步加强。投入资金6.05亿元，完成各类水利工程6.05万处，解决了25.6万农村人口的饮水安全问题，完成了53处病险水库的除险加固；完成通乡公路建设118公里、通畅工程 1415公里 ，新增通畅行政村432个。农业生产综合机械化水平达到51.1%，比上年提高5.2个百分点。农业标准化工作成绩突出。农产品市场准入制度全面实施，农产品质量安全水平得到提高。乡村清洁工程示范点建设任务全面完成。大通湖统筹城乡发展试验区建设扎实推进。

　　（四）积极开展城市创建活动，城市形象有新提升

　　大力推进新型城镇化，城市化率达到41.5%，比上年提高1.75个百分点。以创建国家卫生城市和省级文明城市为抓手，提升城市建设和管理水平。启动了城市特色研究和中心城区控制性详细规划的编制。市规划馆建成并对外开放。市城建投成功发行9亿元企业债券。中心城区市政基础设施建设完成投资24.3亿元，完成了金山北路、康富南路、桃花仑西路、五一西路等道路和95条背街小巷的提质改造及资江一桥的维修工作。中心城区绿化植树十万株，新增绿地面积225.2万平方米，绿化覆盖率达到38%。启动了城市亮化工程，开展了“创卫百日综合整治行动”，市容市貌明显改观。加强供电、供水、供气和公共客运保障，服务水平进一步提升。污水处理“三年行动计划”顺利实施，城北、沅江、南县、桃江四个污水处理厂提前投入运行。垃圾无害化处理场建设稳步推进。全年完成小城镇基础设施建设投资18.9亿元。

　　（五）努力扩大消费需求，消费市场繁荣活跃

　　不断加强市场体系建设，大力推广现代流通方式，积极培育新的消费热点，城乡消费持续活跃。“家电下乡”和“汽车、摩托车下乡”活动成效突出，实现销售额8.5亿元。全市汽车保有量达9.1万辆，新增2.14万辆。房地产业稳步发展，完成房地产投资52亿元，商品房销售额29.9亿元，比上年分别增长11.5%和26.3%。旅游业加快发展，接待国内外游客1142.7万人次，旅游总收入62.8亿元，分别增长32.5%和30.2%；益阳先后被授牌为中国诗歌之乡、中国黑茶之乡。全年保险消费超过20亿元。

　　（六）注重协调发展，社会事业全面进步

　　着力推进科技进步，争取国家和省级科技计划71项。全市有206家企业与高等院校开展了产学研合作。益阳首次成为国家“科技进步合格市”和全国知识产权试点城市。加快教育强市建设，各级各类教育协调发展，“普九”债务化解工作全面完成，义务制教育学校绩效工资改革工作稳步开展，教育保障条件和教育教学质量进一步提高。积极发展公益性文化事业，群众文化活动日益丰富，文化遗产保护利用工作加强，农村文化“四大工程”建设全面推进，文化市场健康有序。医疗卫生体制改革稳步推进，农村卫生体系建设、社区卫生服务中心建设得到加强，疾病防控工作效果明显。广泛开展全民健身运动，群众体育工作持续发展。第七次社区居委会换届选举工作顺利完成。中心城区数字电视整体转换工作按期完成，广播电视事业实现新的跨越发展，益阳日报、益阳门户网等新闻媒体的舆论导向作用充分发挥。人口计生工作继续加强，被评为全省先进单位。加强国土和矿产资源管理，严格执行土地基本国策。统计、档案、保密、民族宗教、外事侨务、台办、科协、地方志、地震、消防、气象、水文、住房公积金、无线电管理、老龄、残疾人、红十字等工作取得新成绩。

　　（七）切实改善民生，社会保持和谐稳定

　　就业再就业工作扎实推进，全市城镇新增就业3.39万人，返乡的农民工基本实现重新就业。“新农保”试点在赫山区正式启动，医疗保险制度实现了全覆盖，城乡低保应保尽保，社会保障体系逐步健全，社会保险待遇进一步提高。完成了658户洞庭湖区捕捞渔民无房户安居工作任务。12所乡镇敬老院、20个村级“五保之家”的改扩建及28所乡镇卫生院建设全部完成。新增廉租住房9802套，城镇廉租住房保障户数达到31730户。完成了农村1300户因灾倒房户的重建和1500户危房改造。对口帮扶安化贫困乡镇工作有序推进，重点贫困村扶贫开发取得进展。抓好精神文明建设，加强了对落实科学发展观和城市文明建设的教育与宣传。重视人民群众来信来访，认真解决上访群众的合理诉求，切实加强法律援助工作，有效化解人民内部矛盾。加强矛盾纠纷的排查调处，积极开展“三调”联动工作，努力防范和稳妥处置各类突发性、群体性事件。全面实施“平安工程”，综治、维稳两项工作全省排名第一。安全生产形势保持平稳。开展产品质量和食品、药品安全专项整治，依法打击传销和制假售假行为，市场经济秩序进一步规范。“五五”普法深入开展，依法治市工作全面推进。认真开展省级“双拥”模范城创建工作，重视全民国防教育，成功举办了防空防灾一体化综合演练，国防动员、后备力量建设和人防工作进一步加强。

　　（八）努力改进政风，政府自身建设得到加强

　　围绕提高政府公信力、执行力，创新政府管理，加强制度建设，办事效率不断提高，政务环境得到改善。认真贯彻《湖南省行政程序规定》，全面推进依法行政。政府机构改革已经启动，交通体制改革和市直水管体制改革基本完成，交通体制改革妥善安置人员2214人。成立市交通发展投资公司，完善了交通建设融资平台。财政体制改革稳步推进，中心城区财政管理体制得到规范，国库集中收付制度改革全面推开。行政审批制度改革不断深化，行政许可项目比上年减少20%。政务公开工作取得成效。市政务中心单位进驻率达88%，项目进驻率达97%。市公共资源交易中心投入运行。政府自觉接受人大及其常委会的法律监督和工作监督，自觉接受政协的民主监督，重大决策主动与政协协商。全年共办理市人大代表建议162件、市政协委员提案239件，办复率为100%。政府部门与各民主党派、工商联的工作联系进一步加强。切实强化审计监督，加强行政效能监察，坚持不懈地抓好纠风治乱和反腐败斗争，勤政廉政建设得到加强。

　　各位代表，过去的一年，在金融危机严重影响、开局比较艰难的情况下，我们战胜了各种困难，圆满完成了市四届人大一次会议确定的目标任务。成绩的取得，得益于中共益阳市委正确判断宏观经济形势，及时采取了一系列有效措施；得益于全市人民和衷共济，团结奋斗；得益于社会各界通力协作，大力支持。在此，我代表市人民政府，向各位人大代表、政协委员，向奋斗在各条战线的全市广大干部群众，向离退休老同志，向各民主党派、工商联、无党派人士、各人民团体，向驻益人民解放军和武警官兵、公安干警，向中央和省属驻益单位，向所有关心、支持和参与益阳建设的各界朋友，表示衷心感谢并致以崇高的敬意！

　　在肯定成绩的同时，我们清醒地认识到前进中的困难和不足。经济总量不大，产业结构不优，特别是工业化水平不高；财政收支矛盾加剧，支出压力加大；创业氛围不浓厚，民间投资不活跃；小城镇规划、建设、管理严重滞后；农民持续增收和统筹城乡发展难度较大；社会事业发展还存在许多薄弱环节；政府工作人员的服务意识、创新意识仍有待增强；发展环境有待进一步优化。这些问题，我们必须高度重视，在今后的工作中切实加以解决。

　　 二、2010年工作的指导思想与奋斗目标

　　2010年是实施“十一五”规划的最后一年，做好今年的工作，直接关系着进一步有效应对国际金融危机影响，巩固经济回升基础，并为“十二五”发展创造良好条件。当前，世界经济出现了明显回暖的积极现象。为继续保持经济平稳较快发展，国家将保持宏观经济政策的连续性和稳定性，继续实施积极的财政政策和适度宽松的货币政策，这有利于我们继续扩大投资、夯实发展基础；国家加大经济结构调整力度，转变发展方式，必将进一步加快沿海产业转移，有利于我们扩大招商引资。省内长株潭“两型”社会试验区和“3+ 5” 城市群建设加速推进，环洞庭湖经济圈发展得到重视，对我市的发展拉力增强，有利于我们更加充分地发挥区位优势。我市多年来打基础的成效逐步显现，特别是一大批重大基础设施和产业项目的建设和投产，进一步增强了我市的发展后劲，我市即将进入新一轮加快发展期。益阳的发展面临诸多有利条件。但是，国际金融危机的影响远未结束，国际贸易保护主义抬头，今年发展的复杂性、不确定性明显增强。我们一定要认真研判形势，及早采取应对措施，坚定信心，抢抓机遇，奋力开创发展新局面。

　　2010年政府工作的指导思想是：坚持以邓小平理论和“三个代表”重要思想为指导，以科学发展观统揽经济社会发展全局，全面贯彻落实党的十七届四中全会和中央、省委、市委经济工作会议精神，紧紧围绕“坚持科学发展，奋力后发赶超，建设绿色益阳”的目标，进一步解放思想、改革创新，优化经济结构，转变发展方式，强力推进项目建设，努力扩大招商引资，扎实推进新型工业化、新型城镇化和现代农业建设，切实加强城乡统筹，全面发展各项社会事业，着力保障和改善民生，实现经济平稳较快发展，确保社会和谐稳定。

　　贯彻落实这一指导思想，必须把继续加快发展作为今年政府工作的首要任务，把新型工业化作为促发展的第一推动力，把扩大投资作为促发展的根本途径，把加快转变发展方式和调整经济结构作为促发展的主攻方向，把统筹城乡发展作为促发展的重要举措，把深化重点领域和关键环节改革、提高对外开放水平作为促发展的强大动力，把改善民生、促进和谐作为促发展的出发点和落脚点，切实提高经济社会发展的质量和水平。

　　2010年经济社会发展的预期目标是：生产总值增长13%以上，财政总收入增长14%以上，全社会固定资产投资增长35%以上，社会消费品零售总额增长18%，进出口总额增长10%，城镇居民人均可支配收入增长10%，农民人均纯收入增长10%，城镇化率提高1.5个百分点，城镇登记失业率控制在4.5%以内，万元生产总值能耗下降4%，万元规模工业增加值能耗下降5.2%，化学需氧量和二氧化硫排放量分别控制在8.3万吨和7.02万吨以下，人口自然增长率控制在7‰以内。

三、努力推动经济又好又快发展

　　 （一）狠抓招商引资与项目建设，进一步增强投资拉动力

　　突出抓好招商引资。开展以“科学招商、实效招商”为主题的招商引资年活动，加强招商信息平台建设，完善全员招商，发挥小分队招商、以商招商等好经验，切实增强招商针对性和计划性。进一步发挥国家“加工贸易产业梯度转移重点承接地”的作用，积极承接产业转移。大力加强装备制造特别是汽车零部件产业和船舶产业、电子信息产业、食品加工业、现代服务业和基础设施建设等方面的招商，十分注重引进战略投资者，吸引世界500强、国内500强、央企和上市公司入驻。精心组织“上海世博会益阳招商推介会”等专题招商活动，抓好“第五届湘商大会”等省级会节招商活动。通过强化责任、做好跟踪服务，加快招商项目落地生根，提高项目履约率。完善招商引资目标管理考核办法，提高招商引资的质量水平。力争全年实际利用市域外资金形成固定资产投资增长20%，实际利用外资增长10%。

　　注重做好立项争资。紧紧抓住各方面的政策机遇，结合编制“十二五”规划，进一步加强项目库建设，精心谋划一批重大产业项目、民生项目和基础设施项目。抓好项目的向上对接，争取更多的项目进入国家、省计划笼子。加强对市直部门立项争资目标任务的考核和落实。

　　全力推进项目建设。深入开展“项目建设年”活动，继续下大力气抓好重点工程建设，确保项目建设快速推进。要突出抓好交通重点工程等基础设施项目建设，加快二广高速、杭瑞高速益阳段、319绕城高速建设，实现S308西姚公路和黄柘公路、S206桃江至横市公路、益宁城际干道沧宁段建成通车，加快G207益阳段、常安高速桃马连接线、S225安化段、沙头大桥、黄茅洲大桥、朝阳汽车站等项目建设进程；力争长益常高速铁路、益马、马溆和益娄高速公路、资江五桥等项目尽快开工；争取常岳九铁路、安张衡铁路、益娄铁路复线等项目的前期工作取得实质性进展；认真实施好国省干线路网改造、主要航道和码头的建设与整治项目。要扎实推进重大产业项目建设，加快汇盛电子科技园等项目的建设和投产进程，力争中海船舶、中核第二三建设公司等项目尽快开工，全力推进桃花江核电站建设。要认真抓好重大民生和社会发展项目建设。启动气象多普勒雷达项目建设，提高对气象灾害的监测、预警和防御能力。加快推进全市“十大信息化工程”。

　　（二）加快重点产业与园区经济发展，强力推进新型工业化

　　突出扶持重点产业和重点企业。加强对重点产业的扶持和培育，进一步做大做强装备制造、电子信息、能源、食品加工等具有比较优势的重点产业，同时继续对接好省“双百工程”、“双十工程”、“四千工程”，扎实推进我市“四个一工程”，帮助一批成长性好的企业快速发展，力争全年新增规模工业企业70家以上。对于装备制造业，重点是支持中联重科、益阳橡塑等行业龙头企业发展；着力打造高新区东部新区汽车零部件基地；抓好以中海船舶、太阳鸟游艇为龙头的益阳船舶工业园建设。对于电子信息产业，要以艾华电子、龙建达电阻、汇盛科技等为重点，抢抓3G产业发展的机遇，继续扩大产业规模。对于能源产业，主要是积极推进金塘冲水电站开工建设，支持晶鑫科技、科力远、中科恒源、漉湖风电等新能源企业加速发展，大力开发我市丰富的水能、风能、生物质能资源，大力发展新能源产业。对于食品加工业，要以克明面业、益华水产、顺祥水产、辣妹子、粒粒晶米业、口口香米业、颐丰食品等农业产业化龙头企业为重点，加快我市传统食品加工企业技术改造，继续扩大品牌影响力。

　　加快工业园区建设。始终把工业园区建设作为推进新型工业化的主战场，摆在战略高度抓紧抓好。高新区东部新区作为“两型”社会先导区，要利用区位和政策优势，继续抓好道路、标准厂房、生活设施等配套基础设施建设，做好鱼形山及周边地区的发展规划，着力引进重大项目，推动产业集聚，尽快打造成为我市重要的经济增长极。高新区、龙岭工业园、长春工业园、沅江经济开发区等已具有一定基础的园区，要进一步完善配套设施，加大招商力度，促进关联产业聚集，形成产业特色。其他省级工业园区和各乡镇工业园，要抓紧完善规划，加快基础设施建设，提高项目承载能力，多方引进项目落户，实现实质性发展。

　　积极推进技术创新。认真对接国家产业政策，加强对传统产业的技术改造，促进优化升级。进一步推动产学研结合，深化企业与高等院校、科研院所的合作。大力支持企业自主创新，开发具有自主知识产权的新技术、新产品、新工艺，促进黑茶生物萃取、金博科技扩产工程、益阳轻型特种装备产业园等高新技术产业项目早日建成投产。力争今年全市省级以上高新技术企业达到40家，年销售收入过亿元的高新技术企业达到15家。扶持科技型中小企业发展壮大。加大人才培养与引进力度，加强职业经理人队伍建设，鼓励高层次专业技术人才进入优势产业、优秀企业和园区。

　　认真抓好节能减排。按照《建设绿色益阳行动纲要》的要求，把握国家发展低碳经济、扩大资源综合利用投资，加大对节能降耗、循环经济、技术改造支持的有利时期，严格落实环境保护、节能减排的各项措施，提升工业经济的“两型”水平。积极推广节能减排新技术，加快实施节能减排重点工程，促进造纸、化工、建材、冶炼等行业污染排放量的有效削减；进一步强化环境监察与目标管理，坚决打击环保违法行为。

　　（三）扎实抓好“三农”工作，加快农业农村经济发展

　　不断改善农村基础设施条件。要按照统筹城乡发展、建设社会主义新农村的战略要求，加强农村基础设施建设。抓好洞庭湖治理、水利工程结合血防灭螺和大型灌排泵站更新改造；启动大通湖东垸、共双茶垸、民主垸围堤加固及安全建设，推进重点中小流域治理；整合资金、统一规划，抓好小型农田水利建设；继续抓好农村饮水安全工程建设和病险水库除险加固；切实加强农村公路和机耕道建设，新建成农村公路800公里。扎实抓好农村配电网建设、农村信息化基础设施建设。进一步抓好农村沼气等可再生能源建设。全面实施农村清洁工程。

　　积极推进农业结构调整。围绕区域优势农产品，大力发展特色产业和品牌农业，走区域化布局、标准化生产、规模化经营、产业化发展的路子。在稳定粮食生产的前提下，引导农民以效益为中心，扩大经济作物种植面积，突出抓好竹木产业、蔬菜产业、生猪产业、水产业、黑茶产业发展和棉花生产，加快规模化、标准化种养小区建设。进一步培育和壮大农业产业化龙头企业，大力发展精深加工，提高农产品附加值。按照“巩固生态体系建设、加快林业产业发展、全面提升市民生态意识”的整体要求，加快国家现代林业示范市建设，全面完成集体林权制度的主体改革，加快推进配套改革。积极推进农村土地流转，探索农村宅基地的流转形式，健全土地流转服务体系。

　　拓宽农民增收渠道。始终把农民增收的问题作为农村工作的重点，落实国家各项强农惠农政策，促进农民持续增收。深入开展涉农收费专项清理和农民负担重点治理，继续减轻农民负担。抓好农村劳动力就业技能培训，提高农民转移就业能力。注重繁荣本地二三产业，更多地吸纳农村劳动力就业。加快发展政策性农业保险，建立农业再保险和巨灾风险分散机制。扶持壮大农机服务组织，推进农机社会化服务。加强农村经纪人培训，促进农民专业合作组织的规范发展，发挥供销合作社在农村流通中的主导作用。加快大型农产品交易市场的规划和建设，重视农产品冷链建设，搞活农产品流通。继续抓好扶贫工作。高度重视库区发展，切实抓好移民开发和金塘冲电站移民安置工作。

　　重视做好农产品质量安全工作。积极推进农业标准化示范县（区）建设。严格农业投入品管理，加大农业行政执法力度，有效净化农资市场。加强农产品检验检测能力建设，抓好农产品质量认证，进一步完善农产品市场准入制，确保农产品的质量安全。加强重大动植物疫病防控，确保不发生重大疫情。

　　（四）抓好城市建设管理与开放发展，加速推进新型城镇化

　　抓好城镇规划修编和规划落实。按照城市发展定位，遵循城市规划与经济布局、生态环境、基础设施相适应的要求，启动我市城市总体规划修编，实现中心城区控制性详细规划全覆盖，基本完成县城和重点镇控制性详细规划。组织完成综合交通等专业规划、各区县（市）新一轮建制镇总体规划和村庄规划。要注重规划的战略性、前瞻性，切实保护山水资源，严格管理土地资源、历史文化资源，体现“绿色益阳”特质，确保可持续发展；同时要注重规划的开放性、协调性，突出对接与融合发展的要求，为加快区域融合、加速城乡一体化预留足够的空间。要抓好规划落实，加大规划执法力度，严格落实市、区、街道、社区（村）四级联动共防共治违法违章建筑办法。

　　加强中心城区建设与管理。以创建国家卫生城市、国家森林城市、省级文明城市活动为抓手，全力实施城市清洁工程、提质改造工程、绿化亮化工程，加强城市基础设施建设，完善城市公共服务设施体系，改善城市形象，提升城市品位。重点抓好桃花仑东路等城市道路提质改造与新建工程；解决中心城区小街小巷的路面建设和亮化问题；继续实施“十万株树进城”工程，提高绿化总体水平；加强城市社区建设，改善社区工作条件。继续推进城市管理综合执法，逐步建立管理长效机制。大力整治城区交通秩序，抓好城中村改造、农贸市场管理、渣土运输管理、占道经营等老大难问题。创新城市建设投融资体制，大力盘活城市资源，特别是提高土地利用效率，多渠道筹措城市建设资金。创新城市供水、供气管理机制，规范市政公用事业特许经营制度。改革完善城市拆迁政策，采用货币补偿等安置模式，既切实维护被拆迁人的利益，又确保城市建设的顺利进行。

　　加快推进县城和小城镇建设。抓住国家当前把推进新型城镇化的重点放在加强中小城市和小城镇发展上的机遇，大力推进小城镇建设。抓好县城扩容提质，加快灰山港、梅城、平口、沧水铺、茅草街、河坝等重点镇发展，构筑布局合理、特色鲜明、产业繁荣的城镇体系，增强城镇对农村的辐射带动力。重视边界城镇建设和经济发展。抓好资阳、赫山两区的污水处理管网建设，推进东部新区、安化污水处理厂和沅江、南县、大通湖、桃江、安化等五个生活垃圾无害化处理项目建成投入使用。统筹抓好小城镇供水、供电、供气、信息等基础设施建设，加快公共服务设施建设，进一步提升城镇公共服务水平。把促进农民在城镇就业和落户作为推进新型城镇化的重要任务，落实城镇户籍管理新办法，加快农村劳动力向城镇稳定转移。

　　（五）扩大消费需求，积极发展现代服务业

　　加快发展旅游业。把旅游业作为支柱产业来打造。围绕建设长沙至张家界旅游线路中转地、长株潭休闲度假目的地，抓好旅游产业发展规划和旅游项目建设。突出产业、产品、文化与旅游相结合，将地域特色文化与景点开发统筹规划，抓好山乡巨变第一村、茶马古道创建4A级景区的工作。加强旅游基础设施建设，促进景区景点提质升级。强化宣传促销，引导和支持旅游企业积极开拓境内外客源市场。加强行业管理，提高行业服务水平。

　　努力促进城乡消费。全面落实国家扩大内需政策，抓紧制定扶持三产业发展的政策措施，促进三产业的大繁荣。加大“家电下乡”、“汽车、摩托车下乡”推广力度，加快推进汽车以旧换新工作。切实加强“双百市场工程”和农产品批发市场标准化建设，加快推进农贸市场的改造升级；扩展社区商业功能，大力推广“便利消费进社区、便民服务进家庭”的“双进工程”。完善对普通自住房和改善性自住房消费的政策扶持，扩大住房公积金覆盖面，积极发展住房公积金个人住房贷款，促进房地产市场健康快速发展。

　　大力发展生产性服务业。以推进铁公水联运体系建设、改善物流条件为重点，加强水运工程建设，支持益阳火车东站和安化火车站建设，启动资阳汽车站、安化城南汽车站的改造，逐步完善流通运输网络。加快中心城区现代物流园区建设，整合一批现有物流企业，初步建立起装备齐全、运行规范的物流体系。积极推进农村邮政物流和城市精益物流业的发展。不断完善中介服务市场，促进中介行业健康发展，培育服务外包、会展等新兴服务业态，带动生产性服务业整体水平的提高。加强风险管理，拓宽保险服务领域。继续发展传统服务业。

　　支持发展对外贸易，加强对外交流与合作。启动海关、商检大楼建设，继续做好检验检疫机构申报，进一步改善通关条件。鼓励和支持大型出口企业提高管理水平，提升产品技术含量。扶持一批有发展潜力的中小出口企业，培育新的出口增长点。积极支持商（协）会建设，提高行业对外交流合作能力。更多地参与区域交流与合作，全面提升对外开放水平。

　　（六）坚持改革创新，增强经济发展活力

　　加大体制改革力度。根据中央和省里的政策要求，按照“循序渐进、稳步推进、综合平衡、统筹兼顾”的原则，推进行政管理体制改革、交通体制改革、文化体制改革、医疗卫生体制改革等各项改革。进一步整合行政资源，加快投融资体制创新，健全投融资平台。稳步推进财税体制改革，全面推动会计集中核算向国库集中支付转轨，巩固税收协控联管工作成果，建立规范有效的财税收入征管长效机制。深化资源环境价格改革。加大国有资产监管力度，提高国有资产经营水平，稳步推进国有企业改革。抓好“一事一议”筹资筹劳奖补试点工作。要特别注重城乡改革联动，力争在统筹城乡公共保障上取得突破。

　　大力激活资本市场。优化金融生态环境，大力发展现代金融业。继续开展好“金融促发展、信贷早春行”银企对接活动。支持省、市担保公司的运作，努力扩大担保范围和份额，积极创造条件引进股份制商业银行和村镇银行，积极稳妥组建小额贷款公司，帮助中小企业解决融资难问题。鼓励企业上市融资，扩大直接融资规模，实现上市公司零的突破。出台相关政策，鼓励民间资本参与重大项目建设。加快推进社会信用体系建设，营造诚实守信的社会环境。

　　推进新一轮全民创业。加强创业文化的培育和宣传，完善和落实鼓励全民创业的各项政策措施，抓好全民创业的培训与指导，激发全民创业热情。积极引导有资金、有技术、懂管理的返乡农民工大胆创业；大力支持高校毕业生、下岗失业人员、军队退役士兵自主创业；引导企业强化创业意识，积极开展“二次创业”。大力发展非公有制经济，支持民营企业做大做强。

四、促进社会全面进步与和谐稳定

　　（ 一）加快发展各项社会事业

　　坚持优先发展教育事业。深入推进教育强市建设，完善教育优先发展的政策措施。加大对教育的投入力度，确保预算内教育经费占财政支出的比例同口径提高2个百分点。加快合格学校建设。健全覆盖城乡的学生资助体系。支持高等院校的建设与发展。加强职业教育基础能力建设，支持国基职教园发展，探索职业教育办学模式改革。推进教育管理和教师人事制度改革，加强教师培训，提高教师队伍素质。落实好教师的政策性待遇，建立健全对农村偏远地区教师的激励机制，营造尊师重教的浓厚氛围。加强学校安全稳定工作，进一步优化教育发展环境。

　　大力发展文化体育事业。加强公共文化服务体系建设，突出社区和乡镇文化设施建设，继续抓好农村文化“四大工程”建设。加快文化产业发展，完成益阳剧院改革试点工作，盘活文化企业。积极推进市群众艺术馆改扩建工程，不断完善市博物馆新馆陈列展示功能。加强文化市场和出版物市场管理。进一步办好《益阳日报》、益阳电视台、益阳门户网和益阳新闻网。规划建设数字影视文化城，继续推进广播电视“村村通”工程和“农村电影数字化”工程。深入开展全民健身活动，同时加强体育人才培养，积极备战省第十一届运动会，进一步加快体育产业发展。

　　健全公共卫生服务体系。不断完善新型农村合作医疗制度，抓好国家基本药物制度、公共卫生与基层医疗卫生事业单位绩效工资等医改试点工作。切实抓好各类传染病、慢性非传染性疾病和职业病等重大疾病的防治工作。进一步健全医疗救治体系，完善突发性公共卫生事件应急机制。加强基层卫生和妇幼卫生工作。强化卫生监督执法，进一步加强行风建设。大力发展中医药事业。积极开展群众性爱国卫生运动。

　　扎实做好人口资源环保等工作。严格执行人口和计划生育工作目标管理责任制，加强综合治理，抓好基层基础工作，加大社会抚养费征收力度，遏制违法生育反弹；严厉打击“两非”行为，降低出生缺陷发生率，提高出生人口素质。加强洞庭湖区和资水流域的环境保护和生态建设，认真推进中心城区第二水源选址工作。加强国土和矿产资源管理，加强基本农田保护，进一步规范土地市场，千方百计保障重点项目建设用地。加强国防动员和后备力量建设，支持武警部队建设，深入开展省级“双拥”模范城创建活动。支持工、青、妇等群团组织依法依规参与社会管理和公共服务，进一步抓好妇女儿童发展规划的实施，确保终极评估验收合格。抓好第六次全国人口普查的组织实施工作。确保档案馆年内竣工。继续做好统计、保密、民族、宗教、外事、侨务、台办、科协、残疾人、地方志、人防、地震、水文、无线电管理等工作。

　　（二）切实改善民生

　　促进和扩大就业。贯彻落实积极的就业政策，继续做好大学毕业生、零就业家庭、关闭破产改制企业职工、上岸定居渔民、农村贫困家庭等就业困难群体的就业援助工作，切实推进返乡农民工等重点群体的就业。加强职业技能培训，优化公共就业服务，重点为本地企业培训和输送合格劳动者，解决劳动者求职难、企业用工难的问题。努力开发社会公益性岗位，拓展就业容量。推进企业工资集体协商制度，加强劳动保障监察执法和劳动争议调解仲裁工作，依法维护劳动者权益。

　　健全社会保障体系。强化社会保险扩面征缴工作。切实抓好赫山区“新农保”试点工作，继续推进城镇居民医疗保险工作，重点推进农民工参加养老保险、商贸服务行业从业人员参加工伤保险工作，切实解决失地农民的社会保障问题。全面解决国有困难企业职工参加职工基本医疗保险问题，积极探索“新农合”与城镇居民医保资源整合、城乡统筹的路子。加强社保基金监管，确保基金安全平稳运行，逐步提高社会保险待遇水平。加强保障性住房建设，切实解决中低收入者和困难群体的住房问题。规范城乡低保，建立健全临时生活救助、医疗救助制度，加强救灾应急能力建设，完善城乡社会救助体系。认真开展红十字初级救护培训。积极发展社会慈善事业，加快发展老龄事业，加强福利服务体系建设。

　　（三）维护社会和谐稳定

　　深入开展文明创建活动。坚持用社会主义核心价值体系引领全市文明创建工作。大力实施市民素质教育、创“五型”机关、当人民满意公仆、和谐社区等八大主题创建活动，弘扬“崇文尚义、通达超越”的益阳城市精神，全面提高城市文明程度和市民文明素质，力争实现省级文明城市的创建目标。坚持以城带乡、城乡互动，深入开展创“十星户”、“五星村”和“文明创建进农家”竞赛活动，进一步加强农村精神文明建设。

　　切实加强安全生产工作。建立健全安全隐患排查治理长效机制，加强基层安监站和监管队伍建设，全面推行精细化监管，逐步建立重大安全隐患和重大危险源电子监控系统，不断提高监管质量。加强安全生产教育培训，严格落实企业安全生产主体责任，严把安全生产行政许可关。切实推进应急救援建设，提高应对事故风险的能力。重视抓好事关民生的产品质量安全工作，强化食品、药品等重点产品的监管。加强消防安全教育，提高市民的消防安全意识，做好消防工作。

　　建立健全维稳长效机制。严格落实维稳工作责任，努力将不稳定因素化解在基层、消除在萌芽状态。加强普法教育，推进依法治市，启动法治城市、法治县（市、区）建设。进一步完善信访工作制度，加快建立县级矛盾纠纷调处中心，有效整合信访、维稳、综治工作力量，切实加大矛盾纠纷排查调处和信访案件办理力度；继续抓好法律援助工作，扎实推进“三调”联动工作。强化社会治安综合治理，抓好基层基础工作，加强城市治安电子监控系统建设，着力整顿治安重点区域和突出问题。提高国家安全意识，时刻防范和从严打击敌对势力的破坏活动。高度重视政府应急管理工作，提高突发事件处置能力。

　　五、大力加强政府自身建设

　　坚持依法行政，建设法治政府。依法行政是推进依法治市的重中之重。要把依法行政作为政府工作的行为准则，始终贯穿于政府决策、执行、监督的全过程。自觉接受人大及其常委会的法律监督和工作监督，执行人大及其常委会的决议、决定，主动接受政协的民主监督，政府重大决策认真听取政协和各民主党派、工商联、人民团体和社会各界意见，继续加强政府部门与各民主党派、工商联的工作联系，办好人大代表议案、建议和政协委员提案。认真贯彻落实《行政许可法》和《湖南省行政程序规定》，严格依照法定权限和程序行使权力。健全行政执法体制，加强行政执法监督，落实行政执法责任制和过错追究制，促进严格执法、公正执法、文明执法。大力推行政务公开，扩大公开范围，丰富公开形式。

　　加快职能转变，建设效能政府。服务是政府的生命。要加快政府职能转变，调整优化机构设置，推进政企分开、政资分开、政事分开、政府与市场中介组织分开，把政府的主要精力更多地集中到社会管理和公共服务上来。深化行政审批制度改革，减少和规范行政审批。加强政务中心建设，抓好电子政务，提高网上办事和在线服务水平与效率，为投资者和人民群众提供高效优质服务。强化效能建设，进一步提高政府和政府部门执行力，抓好政府绩效评估工作，严格行政问责。切实减少和规范评比、达标活动。优质高效地编制好“十二五”规划。

　　大力转变作风，建设为民政府。政府的责任在于保障民生民利。要注重抓基础设施、基础产业、基础工作，谋求长远发展、持续发展。要强化群众利益无小事观念，发扬简洁务实的政风，把精力更多地向基层倾斜，大力推行“一线工作法”，做到调查研究在一线，问题解决在一线，工作落实在一线。要从群众最不满意的事情抓起，从群众最困难的事情帮起，从群众最期盼的事情做起，真诚倾听群众呼声，真情关心群众疾苦。继续坚持为民办实事，逐步完善为民办实事长效机制。

　　坚持从严治政，建设廉洁政府。加强对公务员的培训和教育，提高业务素质和道德修养。坚持“制度防腐”，进一步完善国有资产管理、工程建设、产权交易和政府采购等重点领域的监管制度，加强公共资源交易平台建设；加强行政监察，完善领导干部经济责任审计，加强对财政资金和重大投资项目的审计监督，建立健全“党风廉政舆情在线”网络监督，增强监督合力，确保行政权力在阳光下运行。强化行政成本意识，大力倡导艰苦奋斗、勤俭节约的作风，力戒奢侈和浪费。认真落实党风廉政建设责任制，坚决打击腐败分子，以清正廉洁的政风推动各项工作。

　　各位代表，益阳的后发赶超正处在一个重要的历史关头，我们的责任重大、使命光荣。让我们紧密团结在以胡锦涛同志为总书记的党中央周围，高举中国特色社会主义伟大旗帜，深入贯彻落实科学发展观，在中共益阳市委的坚强领导下，抢抓机遇，锐意进取，为开创益阳科学发展、后发赶超新局面而努力奋斗！
